# Nutritional Strategies for Intestinal Rehabilitation in Children with Short Bowel Syndrome: A Narrative Review

**DOI:** 10.3390/nu18020180

**Published:** 2026-01-06

**Authors:** Inna Spector Cohen, Hadar Moran-Lev, Reut Levi, Hofit Golden, Igor Sukhotnik

**Affiliations:** 1 Pediatric Gastroenterology & Nutrition Institute, Ruth Rappaport Children’s Hospital, Rambam Health Care Campus, Haifa 3109601, Israel; 2 Department of Pediatrics B, Ruth Rappaport Children’s Hospital, Rambam Health Care Campus, Haifa 3109601, Israel; 3 The Ruth and Bruce Rappaport Faculty of Medicine, Technion—Israel Institute of Technology, Haifa 3109601, Israel; 4 Department of Pediatrics and Pediatric Gastroenterology and Nutrition, Tel Aviv Sourasky Medical Center, Tel Aviv 6423906, Israel; 5 Faculty of Medicine, Tel Aviv University, Tel Aviv 69978, Israel; 6 Department of Clinical Nutrition, Rambam Health Care Campus, Haifa 3109601, Israel; 7 Pediatric Surgery, Dana-Dwek Children’s Hospital, Tel Aviv Sourasky Medical Center, Tel Aviv 6423906, Israel

**Keywords:** short bowel syndrome, SBS, intestinal failure, children, nutritional management, intestinal adaptation, enteral nutrition, EN

## Abstract

**Background/Objectives:** Nutritional management is fundamental to intestinal rehabilitation in children with short bowel syndrome (SBS), yet clinical practice remains heterogeneous and largely guided by expert opinion. Enteral nutrition (EN) is the main driver of intestinal adaptation and progression toward enteral autonomy, but optimal strategies vary according to residual bowel anatomy, postoperative phase, and feeding tolerance. This review aimed to synthesize available evidence on nutritional strategies for pediatric SBS, with a focus on EN initiation, advancement, composition, and outcomes. **Methods:** A structured literature search was conducted in MEDLINE (PubMed), Scopus, Web of Science, Cochrane Central Register of Controlled Trials (CENTRAL), SciELO, and Google Scholar for studies published between January 1975 and October 2025. Pediatric clinical studies addressing nutritional management in SBS were eligible. Study selection followed predefined PICO criteria, with independent screening and quality appraisal by two reviewers, in accordance with PRISMA-ScR reporting standards. **Results:** One hundred and thirty pediatric clinical studies were included, the majority of which were observational, with few randomized controlled trials. EN consistently emerged as a key determinant of intestinal adaptation and progression toward enteral autonomy across all phases of SBS. Outcomes were strongly influenced by residual bowel anatomy, presence of the ileocecal valve and colon, and feeding tolerance. Substantial variability was observed in feeding routes, modalities, diet composition, and advancement strategies. **Conclusions:** EN is a cornerstone of intestinal rehabilitation in pediatric SBS; however, current recommendations rely mainly on observational evidence. Prospective multicenter studies are needed to define optimal nutritional strategies and strengthen evidence-based practice.

## 1. Introduction

Intestinal failure (IF) develops when intestinal length or function is insufficient to maintain adequate nutrient absorption, hydration and normal nutritional status [1,2,3]. The American Society for Parenteral and Enteral Nutrition (ASPEN) defines pediatric IF as a reduction in functional intestinal mass below the level required to sustain normal growth, adequate nutrient absorption, or electrolyte balance, resulting in dependence on PN for at least 60 days within a 74-day consecutive period [1,2]. SBS represents the most common cause of IF worldwide, accounting for approximately two-thirds of adult cases and up to 85% of pediatric IF [4,5]. Despite advances in care, SBS remains a major clinical challenge due to its high morbidity, mortality, and substantial socioeconomic impact.

Although SBS is frequently defined according to the length of residual intestine following bowel resection, intestinal functionality is a more important determinant of clinical outcome. Consequently, SBS is increasingly conceptualized as a functional disorder rather than a purely anatomical condition. This perspective is reflected in definitions such as that of the Canadian Association of Pediatric Surgeons, which defines SBS as the need for parenteral nutrition (PN) for more than 42 days following massive intestinal resection or the presence of a residual intestinal length of less than 25% of the expected length for gestational age [6]. Importantly, while most nutrient absorption occurs in the proximal small intestine, the specific segments resected critically influence fluid balance, nutrient absorption, hormonal signaling, and adaptive capacity, thereby shaping both nutritional requirements and long-term prognosis.

**Figure 1:**
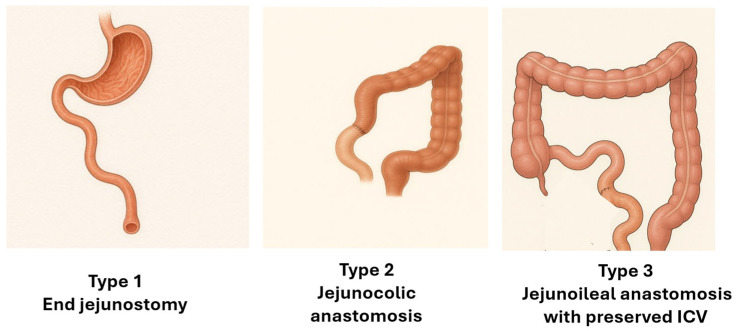
Anatomical types of SBS according to the presence or absence of a small-bowel ostomy, preservation of the ileocecal valve, and colonic continuity.

From a functional standpoint, the anatomical configuration of the remaining bowel is a central determinant of nutritional management and outcomes [7,8] (Figure 1).

One configuration resulting from extensive resection of the small intestine and colon includes loss of the ileocecal valve (ICV), leaving the child with an end-jejunostomy and no colon in continuity (*Type 1-End jejunostomy*). In this situation, loss of ileum—which normally absorbs approximately 80% of intestinal fluid—and loss of the colon, which absorbs the remaining 20% and can augment absorption when needed, lead to rapid intestinal transit, high-output losses, and a high risk of dehydration and electrolyte disturbances [9,10]. The high permeability of the jejunum further exacerbates sodium and fluid losses, particularly when low-sodium or markedly hypo- or hyperosmolar fluids are ingested. Resection of the distal ileum and proximal colon also eliminates enteroendocrine L-cells responsible for the secretion of peptide YY (PYY), glucagon-like peptide-1 (GLP-1), and glucagon-like peptide-2 (GLP-2), which are hormones that are essential for slowing intestinal transit and supporting mucosal adaptation [7,11]. In addition, impaired bile acid reabsorption limits mixed micelle formation and compromises fat absorption [8]. Consequently, many children with this anatomical configuration require prolonged PN to maintain hydration and nutritional status.

A second anatomical configuration is characterized by extensive ileal resection with the remaining jejunum anastomosed to a portion of the colon (*Type 2-Jejuno-colonic anastomosis*). Under normal physiological conditions, the jejunum absorbs most macronutrients and water-soluble vitamins, whereas the ileum exhibits substantial adaptive capacity following jejunal loss. Clinical studies indicate that absorption of protein, carbohydrates, and many vitamins and minerals may be preserved when sufficient healthy ileum remains [12,13]. However, reduced jejunal hormone secretion in this context may decrease pancreaticobiliary stimulation and contribute to gastric hypersecretion, thereby altering the luminal environment [11]. Although the ileum plays a smaller role in macronutrient absorption, its slower transit and greater length confer significant adaptive potential. As in more extensive resections, loss of ileal function impairs bile acid reabsorption and limits mixed micelle formation, adversely affecting fat absorption [8,14].

A third anatomical configuration includes patients who retain a segment of terminal ileum, preservation of the ICV and an intact colon (*Type 3-Jejuno–Ileo–Colonic Continuity*). Beyond small-bowel length, preservation of the colon is increasingly recognized as a critical determinant of outcomes. The colon can markedly increase its absorptive capacity, supporting fluid and electrolyte balance, slowing intestinal transit, and improving energy salvage. Retention of colonic continuity is associated with reduced fecal nutrient losses and a higher likelihood of decreasing PN dependence [15,16,17,18]. Some experts equate preservation of at least half of the colon with maintaining approximately 50 cm of the small intestine, although this relationship varies among patients [16,19]. Colonic fermentation of unabsorbed carbohydrates produces short-chain fatty acids (SCFAs), which provide additional calories and stimulate GLP-2 and PYY release, thereby supporting mucosal adaptation [11,20,21]. When feasible, early restoration of intestinal continuity enhances these physiological benefits and may improve overall energy salvage.

**Figure 2:**
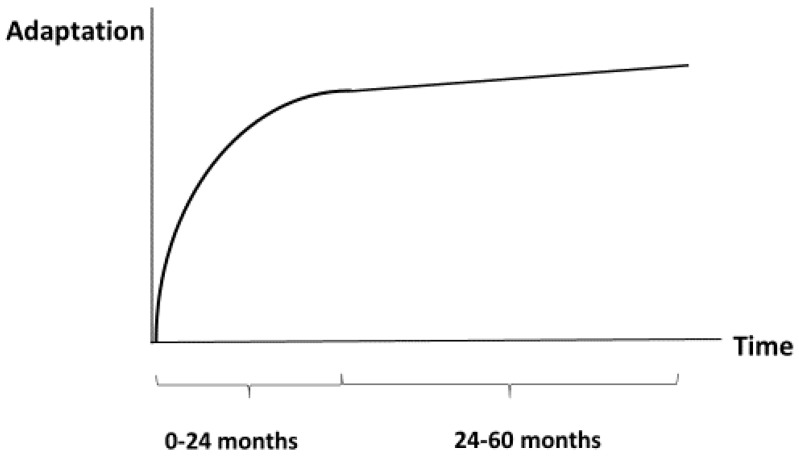
Schematic diagram of development of intestinal adaptation during the first 5 years. Accelerated development during the first 24 months followed by slow development during the next 36 months.

Following intestinal resection, the remaining bowel undergoes a process of intestinal adaptation involving coordinated structural and functional changes that increase absorptive capacity [22]. Adaptation begins within 24–48 h after surgery and may continue for 18–24 months, with many reports suggesting a practical plateau by one to three years in children. Approximately 90–95% of adaptive potential appears to occur during the earlier period, with scope for incremental gains thereafter [22,23,24] (Figure 2).

These adaptive changes include bowel elongation and dilation, increased crypt proliferation with villus hypertrophy, expansion of mucosal surface area, and enhanced enterocyte function, collectively leading to improvements in fluid and electrolyte balance, nutrient absorption and growth [21,22,23,24].

### Postoperative Phases of SBS

**Figure 3:**
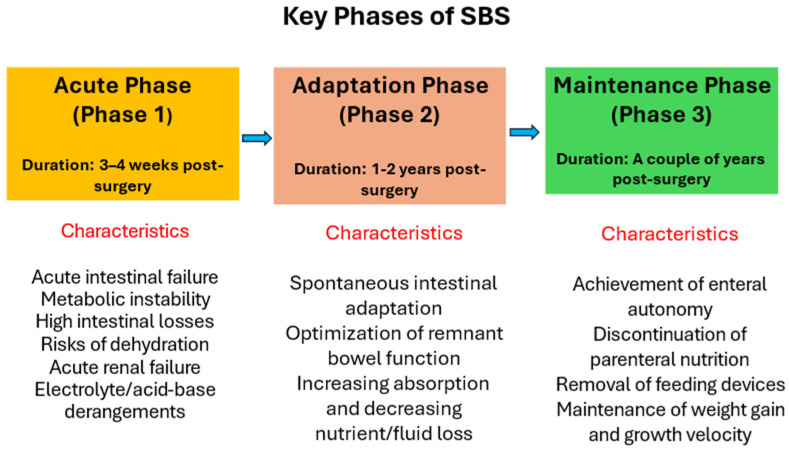
Key postoperative phases of SBS.

Nutritional management of patients with SBS, in both pediatric and adult populations, is commonly described as a phased process that reflects the evolving physiology of the remnant intestine following resection (Figure 3) [2,7,25]. Although these phases are conceptually distinct, they represent a physiological continuum rather than rigid clinical compartments. In practice, nutritional strategies evolve gradually as intestinal function stabilizes, with deliberate overlap between parenteral and enteral nutrition to support adaptation while maintaining metabolic stability.

The initial acute phase begins immediately after surgery and typically extends over the first 3 to 4 weeks. It is characterized by poor absorption of fluids, electrolytes, and nutrients, leading to significant intestinal losses and metabolic disturbances. Gastric hypersecretion is common, in part due to the loss of inhibitory hormones normally released from the terminal ileum, further exacerbating fluid and electrolyte imbalance and reinforcing dependence on PN.

As postoperative stabilization occurs, patients gradually transition into the adaptation phase, which may last one to two years. This phase is characterized by adaptive changes in the remaining small bowel to increase both the absorptive surface (structural adaptation) and the absorptive capacity of isolated enterocytes (functional adaptation), leading to incremental improvements in nutrient and micronutrient absorption. These processes are stimulated by luminal nutrient exposure, gastrointestinal secretions, hormonal signaling from the residual intestine, and the production of peptide growth factors. The extent and pace of adaptation are variable and depend on factors such as residual bowel anatomy, underlying disease, and nutritional management.

The subsequent maintenance phase represents the longer-term stage of intestinal rehabilitation and may extend over several years. Management during this phase focuses on consolidating adaptive gains, optimizing enteral intake, and, when feasible, reducing or discontinuing PN. The overarching goals are achievement of enteral autonomy, removal of feeding devices when possible, and sustained support of growth and growth velocity over time.

PN remains a life-saving therapy for children with SBS, and it is indispensable for maintaining adequate nutrition, growth, and neurodevelopment in the presence of severe malabsorption. Over recent decades, prognosis has improved markedly due to advances in PN formulation, delivery, and monitoring [26]. Nevertheless, prolonged PN exposure is associated with significant complications, including central line–associated bloodstream infections, intestinal failure–associated liver disease (IFALD), vascular thrombosis, metabolic disturbances, and multiorgan dysfunction [27]. To minimize PN-related morbidity, PN should be individualized according to each patient’s tolerated enteral intake and progressively reduced as enteral feeding improves. In clinical practice, PN dependence during the early postoperative period is not viewed as static, but rather as a dynamic phase that progressively gives way to enteral nutrition as intestinal function stabilizes and adaptation begins. Accordingly, management during the acute phase is centered on maintaining metabolic stability with PN while simultaneously identifying opportunities for safe initiation of enteral feeding. This transition marks the first step along a phased rehabilitation pathway, in which PN and EN are deliberately overlapped rather than abruptly exchanged. 

Whenever feasible, enteral nutrition (EN) is preferred because it more closely mimics physiological nutrient delivery [11,28,29]. Beyond its nutritional role, enteral nutrition is the principal driver of intestinal adaptation as sustained luminal nutrient contact exposure stimulates epithelial proliferation and mucosal regrowth [22,23,24]. However, in patients with markedly reduced residual bowel length, overly rapid advancement of enteral feeding may be counterproductive, as excessive feeding has been associated with impaired adaptation and dysmotility [30,31].

Over the past two decades, the epidemiology of pediatric SBS has evolved in parallel with advances in neonatal intensive care, surgical techniques, and intestinal rehabilitation programs, resulting in improved survival and a growing population of children living with chronic intestinal failure. Concurrently, refinements in PN formulation, catheter care, and monitoring have substantially reduced mortality and intestinal transplantation rates, shifting the clinical focus from short-term survival toward long-term outcomes such as growth, neurodevelopment, and quality of life. These improvements have been accompanied by the emergence of multidisciplinary intestinal rehabilitation models, evolving EN strategies, and pharmacologic approaches aimed at enhancing intestinal adaptation, collectively expanding the therapeutic landscape for children with SBS.

Despite these advances, important knowledge gaps and areas of clinical controversy persist. The pediatric evidence base informing nutritional management remains dominated by retrospective studies, small heterogeneous cohorts, and expert consensus, with a marked scarcity of randomized controlled trials. Key unresolved questions include the optimal timing and rate of EN advancement, the most appropriate feeding route and modality, and the ideal composition of enteral diets across different anatomical phenotypes of SBS. In addition, clinical outcomes are frequently not stratified according to residual bowel anatomy or stage of adaptation, limiting the ability to individualize nutritional strategies based on factors known to influence enteral autonomy. As a result, substantial variation in nutritional practices exists across centers.

In this context, a structured synthesis of the available literature is needed to integrate evolving epidemiologic trends, summarize recent therapeutic advances, critically examine areas of limited or conflicting evidence, and identify priorities for future research. This review aims to address these gaps by focusing on the physiological principles underlying nutritional management in pediatric SBS, with particular emphasis on enteral nutrition strategies tailored to anatomical configuration and phase of intestinal rehabilitation.

## 2. Methods

A structured, comprehensive literature search was conducted using the MEDLINE (PubMed), Scopus, Web of Science, Cochrane Central Register of Controlled Trials (CENTRAL), Scientific Electronic Library Online (SciELO), and Google Scholar databases for studies published between January 1975 and October 2025. In addition, a manual search of the reference lists of the retrieved publications was performed to identify additional relevant studies. The objective was to identify all human studies (clinical settings) in children directly addressing nutrition or specified nutrients in relation to SBS. Search terms included various combinations of the following keywords: “short bowel syndrome,” “intestinal failure,” “children,” “enteral nutrition,” “enteral feeding”, “parenteral nutrition weaning,” “phases of SBS,” “types of SBS,” and “intestinal adaptation.” Eligibility criteria were based on the PICO (Population, Intervention, Comparator, and Outcome) framework and were defined according to the inclusion and exclusion criteria summarized in Table 1.

Two authors independently screened the titles and abstracts of all identified studies. Full-text papers meeting the inclusion criteria—including analytical cross-sectional studies, prospective cohort studies, case–control studies, longitudinal studies, randomized controlled trials, case series, and retrospective cross-sectional studies—were retrieved for detailed assessment. Studies were excluded if they did not meet the review objectives, were not in English, or consisted of editorials, letters to the editor, conference abstracts, duplicate entries, or articles without freely available abstracts.

The following key questions were formulated for each phase of SBS (acute, adaptation, and maintenance phases):

- At what time should enteral nutrition (EN) be initiated? How should EN be advanced and administered?
- What type of EN should be used (focusing on specific macronutrients and micronutrients) during each post-surgical phase?
- What are the features of nutritional management depending on the type of SBS (e.g., site of resection and colon-in-continuity)?

To minimize bias at all stages of the review, several methodological safeguards were implemented: Independent screening: Two authors independently conducted literature screening, data extraction, and quality assessment. Discrepancies were resolved by discussion or consultation with a third reviewer. Duplicate removal and data verification: All datasets were cross-checked to avoid duplicate inclusion and confirm data accuracy. Reporting standards: The review was conducted and reported following the Preferred Reporting Items for Systematic Reviews and Meta-Analyses Extension for Scoping Reviews (PRISMA-ScR) guidelines, ensuring methodological transparency and reproducibility [32]. Transparent inclusion criteria: The predefined PICO framework ensured objective selection and consistent eligibility decisions (Table 1). Quality appraisal: The STROBE Statement checklist was applied to cohort, case–control, and cross-sectional studies [33], and the Joanna Briggs Institute (JBI) checklist was used for systematic reviews and meta-analyses [34].

### Definitions

The target population (referred to as children) included neonates, infants, and older children diagnosed with SBS. EN was defined as the delivery of nutrients to the gastrointestinal tract via oral or enteral tube feeding (nasoenteral, gastrostomy, or jejunostomy). Continuous administration typically employs an enteral infusion pump, with the hourly rate calculated by dividing the daily nutritional requirement by the total infusion time. Bolus feed volumes are determined by dividing the total daily requirement by the number of feedings. Minimal enteral feeding was defined as ≤12 mL/kg/day or <25 kcal/kg/day for 5–10 days in infants [35]. Extensively hydrolyzed (semi-elemental) formulas (EHFs) contain hydrolyzed proteins (oligopeptides and free amino acids). Amino-acid-based (elemental) formulas (AAFs) are similar but contain only free amino acids as the protein source. Polymeric (standard) formulas consist of intact proteins [36]. SBS was defined as the need for PN for more than six weeks following massive intestinal resection or a residual intestinal length of <25% of the expected value for gestational age [1,3]. Enteral autonomy (EA) was defined as an achievement of adequate weight and height gain, which are maintained using EN alone for more than three consecutive months [1]. Tolerance was defined as the ability to increase enteral or oral feeds without significant vomiting, diarrhea, or other signs of malabsorption.

## 3. Results

The literature search identified 133 pediatric clinical effect studies that had investigated the effects of different aspects of feeding strategies on clinical outcomes in children with SBS. Very few high-quality clinical randomized control studies are available on aspects of EN in children with SBS because of the relatively low incidence of the disorder and high variety of its manifestations in every patient. Consequently, dietary management is highly variable and depends on both clinical situation and Institutional policy. Five studies covered the timing of initiation of EN. Twenty-five papers covered method of administration (route of delivery). Forty-six studies covered types of nutrition (in the first phase, 38 papers; in the second phase, 20 papers; in the third phase, 7 papers). Ten studies were found on the advancement of EN (in first phase-3 papers, in second phase-5 papers, in third phase-2 papers). Eight papers focused on specific nutrients in relation to SBS (preventing hyperoxaluria, preventing osmotic diarrhea, etc.). In addition, seven case reports and narrative review articles on feeding strategies in children with SBS were identified. Findings from those articles, if relevant, are listed under each key question.

## 4. Discussion

SBS in children is a dynamic condition in which nutritional management must evolve alongside changes in intestinal function. Although postoperative phases provide a useful framework, clinical care rarely follows rigid boundaries, and decisions regarding parenteral and enteral nutrition must be continuously adjusted to balance nutritional adequacy with stimulation of intestinal adaptation. Within this context, the following sections present practical, phase-specific approaches to enteral nutrition initiation, advancement, and composition, focusing on the progressive optimization of enteral feeding as intestinal function improves and parenteral support is gradually reduced, rather than on abrupt nutritional transitions.

### 4.1. Enteral Nutrition at the First Phase of SBS (Acute Phase, Typically Lasts Weeks to Months)

The acute phase begins immediately postoperatively, before intestinal adaptation has occurred, when absorption is markedly impaired. During this period, delayed transit, reduced motility, diarrhea, and substantial fluid and electrolyte losses are frequent, increasing the risk of dehydration [37]. The acute phase typically lasts several days to a few weeks and is characterized by postoperative ileus and high gastrointestinal output [4]. The ESPGHAN Committee on Nutrition further delineates three stages within this phase: an early period (first 24–48 h), an intermediate postoperative period (up to ~1 week), and a recovery period (from ~day 7 until stabilization or transfer from intensive care) [38]. Throughout the early and intermediate stages, clinicians should prioritize hydration and sodium balance, with close monitoring and replacement of losses using parenteral solutions that provide adequate sodium, recognizing that enteral tolerance is generally limited [39].

#### 4.1.1. Parenteral Nutrition (PN)

PN should be initiated early as the default nutritional backbone after major resection, with the goals of preventing acute malnutrition, maintaining fluid and electrolyte homeostasis, and supporting growth while enteral intake cannot meet requirements. In infants after extensive resection, PN is typically initiated within 48–72 h postoperatively [39]. Clinicians should target energy and protein provision consistent with published estimates (preterm: ~60–80 kcal/kg/day; term: ~45–70 kcal/kg/day; amino acids ≥ 1.3–1.5 g/kg/day to sustain positive nitrogen balance) [39]. Clinicians should individualize fluids and electrolytes to measure gastrointestinal and stoma losses and provide routine micronutrient supplementation given the frequency of deficiencies [39,40]. Monitoring should be operationalized as a checklist: daily weights, strict intake/output, serial plasma sodium, and daily urinary sodium, with prompt correction of ongoing losses using appropriate sodium-containing solutions [39].

#### 4.1.2. Timing of EN Initiation

Early EN promotes intestinal adaptation from the acute phase by providing luminal nutrients that stimulate mucosal hyperplasia, support epithelial growth and absorptive capacity. It also enhances secretion of gastrointestinal and pancreaticobiliary hormones that drive gallbladder contraction, digestion, and intestinal motility [27,41]. EN helps reduce PN-associated cholestasis, supports microbiome development—particularly in preterm and high-risk infants—and protects the mucosa from stress-related injury [39,42]. Additionally, EN is simpler, safer, and more cost-effective than PN [36]. EN should be started as soon as clinically feasible to provide luminal stimulation that supports adaptation and intestinal function [39,42]. In practice, initiation criteria vary; therefore, centers should define a local trigger set. Examples include starting once stool passage is documented [43], or using more conservative criteria, such as clear, non-bilious tube drainage; audible bowel sounds; a soft, non-tender abdomen [44].

As infants transition into the late acute phase, EN should be advanced gradually with explicit recognition of persistent malabsorption due to reduced surface area. Typical targets are ~90–120 kcal/kg/day for preterm infants and ~75–85 kcal/kg/day for term infants, with routine monitoring and supplementation for micronutrient deficiencies [39]. To reduce risk of IFALD, clinicians should introduce PN cycling when tolerated once the infant is clinically stable and ≥4 kg—to reduce hypoglycemia risk during PN-free intervals. Avoid premature PN reduction or forced EN escalation that outpaces tolerance [37,39].

#### 4.1.3. Route of Delivery

##### Oral vs. Tube Feeding

When feasible, clinicians should encourage oral feeding early because it provides physiological stimulation (including salivary epidermal growth factor release), supports normal gastric and pancreatic secretions and promotes release of trophic enteral factors and hormones [39,44,45]. Early oral experiences may also facilitate the development of feeding skills and reduce the risk of later oral aversion, a complication that can delay transition from PN and adversely affect quality of life (QOL) [45]. When oral feeding is not feasible (e.g., mechanical ventilation, instability, immaturity, or dysfunction), early initiation of tube feeding is appropriate and preferable to delaying luminal nutrient exposure, to support adaptation and intestinal function.

#### 4.1.4. Gastric vs. Post-Pyloric Feeding

Gastric feeding should be treated as the default route because it is physiological, technically simpler, and can be given as intermittent boluses or continuous infusions, facilitating flexible strategies. Gastric feeding increases nutrient–mucosa contact and supports motility; in SBS, it has been associated with improved transit and reduced diarrhea, thereby promoting adaptation [40,46]. Alternating feeding and fasting—together with cyclic PN—may avoid continuous insulin secretion and limit excessive fat synthesis and deposition [11,37]. Post-pyloric feeding should be reserved for defined indications, such as significant gastroesophageal reflux/aspiration risk or severe gastric dysfunction or anatomical barriers [47]. Clinicians should explicitly weigh trade-offs: post-pyloric access is more technically demanding, costlier, and carries higher mechanical complication risk; it bypasses gastric/duodenal stimulation of biliary and pancreatic secretions and has been linked to intussusception [46,48,49]. Because post-pyloric feeding bypasses the stomach’s regulatory function in controlling the rate of nutrient entry into the small intestine, it must be administered continuously to prevent rapid intestinal delivery and dumping syndrome. Because post-pyloric feeding bypasses the stomach’s regulatory function in controlling the rate of nutrient entry into the small intestine, clinicians should deliver feeds continuously to avoid rapid intestinal delivery and dumping syndrome. This requirement further limits mobility and may affect QOL [44,47]. Current evidence does not show a clear advantage of post-pyloric over gastric feeding in pediatric SBS; practice varies, and ESPGHAN recommends individualized decision-making with gastric feeding as the default unless specific indications favor post-pyloric access [41,43,44,47].

#### 4.1.5. Feeding Modality: Bolus vs. Continuous

Tube-based EN may be given as intermittent bolus, continuous, or mixed regimens. Clinicians should select the modality based on tolerance and practical goals, acknowledging limited comparative pediatric evidence. Continuous feeding can be used to reduce stool output and improve tolerance by decreasing the osmotic load and to improve nutrient absorption by prolonging luminal exposure and facilitating transporter saturation along the intestinal mucosa [27,50], but clinicians should monitor for downsides, including reduced fasting periods, with potential risk of dysmotility, small bowel bacterial overgrowth (SBBO), and IFALD [30,37,44]. No randomized trials directly compare continuous vs. bolus EN in children with SBS; limited observational data suggest continuous feeding can improve tolerance, reduce stool volume, and support weight gain [51,52,53]. Adult SBS data indicate improved overall absorption with continuous vs. oral/bolus feeding [54]. Bolus feeding mimics physiological meal–fast cycles, supporting normal gastric filling/emptying and stimulating motility; it may also promote bile flow and avoid continuous insulin secretion, potentially reducing hepatic steatosis and excess fat accretion [27,37]. In preterm infants, bolus feeding has been linked to better growth and tolerance [55], yet a meta-analysis did not show a consistent overall benefit over continuous feeding [56]. Given limited evidence, randomized trials in pediatric SBS are lacking and centers should operationalize a pragmatic approach: start with the modality that best matches reflux, stool/stoma pattern, and caregiver feasibility, then switch if intolerance (e.g., escalating output, vomiting, poor growth) develops [37,44].

#### 4.1.6. Choice of Feed

International guidelines (ASPEN, ESPGHAN, NASPGHAN, AGA) recommend human breast milk as first-line EN for infants with SBS-associated IF [39,40,50]. This recommendation is grounded in the unique biological composition of human milk (HM), which provides long-chain fatty acids, free amino acids, growth factors, immunoglobulins, leukocytes, bioactive proteins (e.g., lactoferrin, lysozyme), cytokines, hormones, such as epidermal growth factor (EGF), antimicrobial agents, immune and stem cells, and prebiotic human milk oligosaccharides (HMOs). Collectively, these components support mucosal repair and growth, immune function, microbiome development, and neurodevelopment [27,39,44,57,58]. This consensus is reflected in clinical practice: an ERNICA survey reported that 23 of 24 European centers use maternal milk as the primary feeding strategy for infants with SBS-IF [43]. Observational data further associate HM exposure with reduced necrotizing enterocolitis (NEC) risk in preterm infants [58], shorter PN duration [17], and a lower incidence of IFALD [59]. However, these associations are largely derived from observational cohorts, and causality cannot be firmly established, particularly in infants with severe SBS who differ substantially from preterm populations without extensive bowel resection.

When maternal human milk is unavailable, clinicians may consider donor human milk (DHM) for high-risk infants, including those with SBS-IF [28,58]. In very preterm and very low birth weight infants, DHM has been shown to reduce NEC risk by approximately 50% compared with formula [60]; however, whether this protective effect applies to infants with extensive bowel resection remains uncertain, as these populations differ substantially in intestinal anatomy, absorptive capacity, and nutritional requirements.

Growth may be slower due to reduced protein/energy content following pasteurization, necessitating targeted fortification. If DHM is used, clinicians should anticipate lower protein/energy after pasteurization and implement targeted fortification, while acknowledging that SBS-specific evidence remains limited [61]. Evidence supporting this approach in pediatric SBS remains limited. In selected surgical populations, such as infants with small bowel atresia or gastroschisis, DHM has been associated with shorter hospitalization and fewer central line days [62], but these observations derive from small cohorts and may not be generalizable to more severe SBS phenotypes. Pasteurization reduces the activity of several heat-labile components, including bile salt–stimulated lipase, lactoferrin, lysozyme, and some immunoglobulins [57,63,64,65]; nevertheless, DHM appears to retain biological properties not replicated by formula, particularly with respect to intestinal barrier maturation and microbiome profiles more closely resembling those of breastfed infants [66,67]. HMOs, such as 2′-fucosyllactose, may further augment adaptive responses after bowel resection [68], although direct clinical evidence supporting this effect in SBS remains sparse. In contrast, infant formulas lack the complex immunologic and trophic factors present in human milk and contain three- to five-fold lower concentrations of HMOs, resulting in markedly different compositional profiles [69,70]. Although DHM from accredited milk banks is considered low risk, with no serious adverse events causally linked in large biovigilance datasets [71,72], safety does not equate to nutritional adequacy. Importantly, pasteurization reduces protein content by up to 12% and fat content by up to 25% [73,74], and because most DHM is produced by mothers of healthy term infants, its composition may be suboptimal for preterm infants with SBS—including those with NEC. Treating DHM is used in preterm infants with SBS (including NEC) as a high-monitoring strategy requiring individualized fortification and close growth/nutritional follow-up.

Evidence guiding the use of HM fortifiers in SBS is limited and conflicting. Fortification increases osmolarity [75,76,77], which may worsen feeding intolerance or may precipitate metabolic acidosis in infants with compromised mucosal integrity.

If HM is unavailable or not tolerated, clinicians should proceed to infant formula selection using a trial-and-monitor approach, because optimal protein composition remains uncertain [37]. Animal models suggest polymeric formulas may exacerbate diarrhea and prolong PN dependence [78], whereas a single clinical report associated AAFs with shorter PN duration [17]. In contrast, a randomized clinical trial comparing hydrolyzed and non-hydrolyzed protein formulas found no differences in growth, energy intake, or nitrogen balance [14]. Furthermore, a comparative review has not demonstrated differences in time in enteral autonomy (EN) or PN duration among regimens based on extensively hydrolyzed formulas, AAFs, or combinations with human milk [19]. These inconsistent findings likely reflect heterogeneity in patient anatomy, disease severity, timing of intervention, and outcome definitions rather than true superiority of one protein source.

More complex proteins may theoretically enhance trophic signaling; however, reports describing possible associations between SBS and food-related immune responses—including cow’s milk protein allergy (CMPA) [79,80] and noninfectious eosinophilic colitis [11,81,82]—have raised concerns. Available evidence remains limited: only two studies have documented IgE-mediated CMPA in three pediatric SBS patients [79,80]. Many postoperative gastrointestinal symptoms may reflect intolerance rather than true immune-mediated allergy [83], and routine avoidance of complex proteins is therefore not supported by current evidence. Instead, individualized trials with careful monitoring are appropriate. Although most EHF are lactose-free, which may be advantageous in infants with proximal jejunal involvement and reduced lactase activity [44,84], the clinical relevance of routine lactose avoidance remains uncertain. Adult SBS studies demonstrate similar tolerance between lactose-free diets and diets containing modest lactose amounts (up to 20 g/day) [85,86], though extrapolation to infants during early adaptation should be approached cautiously.

The role of dietary fat composition remains similarly controversial. Medium-chain triglycerides (MCTs) may improve fat absorption in settings of rapid transit, bile acid depletion, or SBBO [44]; however, evidence is mixed. In one randomized trial, high-MCT formulas improved fat absorption in patients with colonic continuity but caused osmotic diarrhea in those without a colon [87]. Animal data suggest that long-chain triglycerides (LCTs) may more effectively stimulate intestinal adaptation than MCTs [88], raising concern that very high-MCT formulas could blunt adaptive signaling. Consequently, selection of formula type and fat composition should be individualized based on anatomy (e.g., presence of colon), stool characteristics, growth requirements, tolerance, and center experience rather than applied uniformly.

#### 4.1.7. Advancement of Enteral Feeding

Early feeding practices vary widely across centers [43]. Reported bolus advancement rates range from minimal incremental increases (+1 mL/feed every 12 h in neonates [46]) to more rapid daily volume escalations (10–20 mL/kg/day [40,44]), while continuous feeding protocols vary similarly (from +1 mL/h twice weekly [42] to +1–2 mL/h every 12 h [45]). In clinical practice, EN advancement should follow a tolerance-based algorithm rather than fixed volume targets. Clinicians should define tolerance using stool consistency, stoma output, stool frequency, emesis, hydration status, and early growth trajectory as primary metrics [39,42,43,87].

Proposed thresholds for acceptable stool output vary substantially from ~20 mL/kg/day (≈6 stools/day) to 40–50 mL/kg/day (≈10 stools/day) [39], underscoring the lack of standardized cutoffs; therefore, trends over time should guide decisions rather than single measurements. Decisions regarding feeding rate and route should incorporate intestinal anatomy, gastroesophageal reflux or vomiting, stool/stoma patterns, and presence of feeding tubes [44]. Tolerance should be reassessed at least every 24 h and after any change in feeding volume or formulation, including stool volume/consistency, bloating, and any blood or mucus [31]; feeds should generally not be advanced more than twice within that interval. Pharmacologic adjuncts (e.g., prokinetics, antidiarrheals) may improve tolerance in selected cases [44], but comparative data are limited. During the acute phase, clinical decisions should prioritize physiological tolerance and metabolic stability rather than assumptions regarding formula composition.

As postoperative ileus resolves and intestinal losses stabilize, clinicians should shift from PN-dominant stabilization toward active promotion of adaptation, using EN as the main modifiable driver. Manage this transition as a continuum: increase EN in stepwise increments while titrating PN down proportionally, maintaining growth and biochemical stability rather than adhering to a predefined time point.

### 4.2. Enteral Nutrition at the Second Phase of SBS (Acute Phase, Typically Lasts Weeks to 18 Months)

During the second phase of intestinal rehabilitation, nutritional management aims to gradually increase EN while reducing PN and promoting intestinal adaptation [26,27,28]. Introduction of solid foods at 4–6 months corrected age supports oral motor development and may reduce feeding aversion [89], with paced progression based on tolerance [8]. Strategies that slow intestinal transit may improve absorption [44], particularly in patients with preserved colonic continuity. Nutritional planning must account for residual anatomy, including jejunum, ileum and colon length [44].

#### 4.2.1. Role of PN and Rationale for Progressive EN

Within this phased framework, PN and EN should not be viewed as competing modalities but as complementary components of intestinal rehabilitation, with their relative contributions evolving over time in response to tolerance, anatomy, and adaptive capacity. PN remains essential for growth and neurodevelopment during severe malabsorption; it has contributed to improved survival over recent decades [26]. Nonetheless, its prolonged exposure is associated with significant complications, including CLABSIs, IFALD, vascular thrombosis, metabolic abnormalities, and organ dysfunction [26]. Accordingly, PN should be titrated to enteral tolerance and reduced gradually as EN advances. Rapid, forced advancement of EN—particularly in children with markedly reduced bowel length—should be avoided, as it may impair adaptation and precipitate dysmotility. Decisions to advance EN or reduce PN should be reassessed frequently and guided by objective tolerance metrics, including stool or stoma output, weight trajectory, and electrolyte stability [8,30,31].

#### 4.2.2. Type of Enteral Nutrition

During the second phase of intestinal rehabilitation, EN aims to provide sufficient fluids, electrolytes, and calories to support growth and neurodevelopment while promoting adaptation [44]. However, despite the central role of EN in SBS management, high-quality comparative evidence remains limited, and most recommendations are derived from expert consensus and institutional experience rather than standardized, evidence-based protocols [27,44]. As a result, considerable variability exists among centers with respect to dietary composition and feeding strategies.

#### 4.2.3. Lipid Composition

Fat malabsorption is common in SBS due to reduced surface area, bile acid depletion, and relative pancreatic insufficiency. Experimental and clinical studies suggest that early introduction of a high-fat diet after bowel resection promotes mucosal growth and villus elongation [90,91], whereas low-fat diets have been associated with impaired adaptation and suboptimal weight gain [92,93]. At the same time, high dietary fat intake is not universally beneficial. MCTs may improve fat absorption in the setting of cholestasis or pancreatic insufficiency because they do not require micellar solubilization and enter the portal system directly; however, formulas with high proportion of MCTs (>5% of total fat) have been associated with increased stool output and limited advancement of enteral feeds [44]. Moreover, while higher fat intake allows delivery of adequate calories without increasing feed volume [27], it may exacerbate fecal losses of calcium, magnesium, copper, and zinc [94]. In patients with an intact colon, unabsorbed free fatty acids can enhance oxalate absorption [95], increasing the risk of hyperoxaluria and renal complications [36,44]. Clinically, lipid composition should be selected based on anatomy and stool response: formulas with moderate fat content and mixed LCT/MCT profiles should be trialed first, with avoidance of high-MCT formulations in patients without colonic continuity or with worsening stool losses.

#### 4.2.4. Carbohydrate Quality

Simple sugars are known to exacerbate osmotic diarrhea in SBS and are generally limited [36]. Starch-based carbohydrates are often better tolerated. In clinical practice, both artificial sweeteners and naturally occurring simple sugars have been consistently observed to increase stool output, whether consumed as part of foods or present in compounded medications [36,44]. In contrast, tolerance to lactose is more variable. Children with proximal jejunal resection are more prone to lactose intolerance due to reduced lactase activity, and if symptoms occur, lactose-free or dairy-free alternatives can be recommended [36]. This variability underscores the need for careful, patient-specific assessment rather than uniform carbohydrate restriction. Feeding intolerance should be reassessed after each carbohydrate modification. Clinicians should define intolerance by using objective measures (stool/stoma output, emesis, hydration status, and early growth trends) and avoid assuming superiority of any carbohydrate source in the absence of a consistent clinical response.

Soluble fibers (such as pectin or guar gum) may improve feeding tolerance by increasing viscosity, slowing intestinal transit, and serving as substrates for short-chain fatty acid (SCFA) production. Limited pediatric case series suggest potential benefits in stool consistency and nitrogen absorption [44,45,96,97]. However, these findings are not supported by controlled trials, and fiber supplementation may be poorly tolerated in children without colonic continuity, where increased stool bulk and gas production can complicate ostomy management [36]. Thus, fiber use must be cautiously individualized.

#### 4.2.5. Electrolyte and Fluid Considerations in Older Infants/Children

Fluid and electrolyte management is critical and often underrecognized as a determinant of enteral success. In this population, absorption of free water is limited by rapid intestinal transit and reduced mucosal surface area. As a result, intake of hypotonic fluids alone is often insufficient to correct dehydration and may be associated with increased stool or stoma output, based on physiological principles and clinical experience. Accordingly, overall fluid intake should be carefully titrated to the minimum necessary to maintain hydration. Oral rehydration solutions (ORS) are physiologically preferable because they exploit sodium–glucose cotransport to enhance water absorption; however, their effectiveness in practice may be limited by poor palatability and adherence. ORS should provide 20–25 g carbohydrate, 45–80 mEq/L sodium and have an osmolarity of approximately 300 mOsm [36]. Separating fluid intake from meals is commonly recommended to reduce nutrient losses related to accelerated transit, although this approach is supported primarily by physiological rationale rather than controlled clinical trials. To limit excess intake, some centers may offer ice chips or frozen ORS/electrolyte popsicles [44]. Hyper-osmolar drinks (e.g., juice, soda, sweetened tea) should be avoided as they are associated with increased stool output and a higher risk of dehydration [36].

Children with IF—especially those with stomas—are prone to total body sodium depletion [36,96,97,98], which can impair weight gain [98]. Low urine sodium concentrations (<10–30 mmol/L in a random urine sample) [36] may occur despite normal serum sodium to improve growth [99]. Therefore, close monitoring of stoma output and sodium balance, including periodic urine sodium assessment, is essential [7,100].

#### 4.2.6. Hyperoxaluria

Management of hyperoxaluria illustrates the inherent trade-offs in dietary manipulation. In patients with ileal resection and preserved colonic continuity, a low-oxalate, low-fat diet with high fluid intake is generally recommended to reduce the risk of hyperoxaluria [101,102]. However, in practice, strict dietary oxalate restriction is often difficult to implement. Many foods that are relatively high in oxalate—such as celery, spinach, eggplant, green beans, okra, wax beans—as well as several soluble, high-fiber legumes including chickpeas, green beans, kidney beans, and lentils, are also low in simple sugars and therefore commonly recommended in dietary management of SBS [36,44]. This overlap complicates dietary counselling and may lead to competing nutritional priorities. As noted earlier, dietary fat supports intestinal adaptation and should not be overly restricted, while fluid intake should be limited to prevent worsening diarrhea, yet remain sufficient to avoid dehydration [102]. Achieving this balance requires close and ongoing clinical monitoring. Pharmacologic approaches such as potassium citrate may reduce kidney stone formation but can worsen stool output [4]. Enteral calcium supplementation, which binds oxalate in the colon and reduces its absorption, represents a more practical and physiologically grounded strategy, although careful monitoring remains essential [45,101,102,103]. Additionally, because oral nutrition supplements and enteral formulas contain variable oxalate levels, attention to their oxalate content is warranted when selecting products for children with SBS [102].

#### 4.2.7. Blenderized Feeds

Blenderized tube feeds (BTFs) and real-food–based formulas have gained increasing attention. Retrospective studies suggest potential improvements in gastrointestinal tolerance (e.g., decreased reflux, gagging, retching, and feeding aversion), fewer hospitalizations for respiratory illnesses, and intestinal microbiome diversity [104]. However, evidence for their use in SBS is limited and inconsistent. In a cohort of 58 children with SBS, of whom fewer than 10% were receiving PN, the introduction of BTFs to provide at least 5% of caloric intake was associated with improved stool consistency and gastrointestinal symptoms [105]. Given the relatively small proportion of calories derived from BTFs, it is difficult to determine whether the observed benefits reflect the true effect of blenderized feeds themselves or are attributable to other concurrent dietary or clinical factors. Another small study of 12 children with intestinal failure reported a reduction in PN requirements after transitioning from amino acid or hydrolyzed formulas to BTFs [106]; however, this study was also retrospective and uncontrolled. Practical concerns—including preparation burden, short shelf life, tube clogging, nutrient variability, and the need for specialized dietitian oversight—further limit generalizability. Tube feeding formulas containing real food ingredients (TFRF) may offer a more standardized alternative, but available data are similarly limited to small retrospective series [107].

### 4.3. Enteral Nutrition at the Third Phase of SBS (Maintenance Phase, Typically Lasts from 18–24 Months to Several Years)

The principal goal during the third phase of intestinal rehabilitation for children with SBS is the achievement of EA. In practice, weaning from PN is a prolonged and complex process that may extend over several years and is rarely linear. Readiness to initiate PN weaning is typically determined by a combination of clinical stability, nutritional adequacy, and biochemical parameters, with the overarching aim of ensuring sustained growth and metabolic homeostasis through enteral or oral intake alone. However, no universally accepted criteria exist, and decisions are largely guided by expert judgment rather than validated algorithms.

Multiple factors have been associated with the likelihood of achieving EA in children with SBS. Residual small-bowel length and quality, preservation of the ICV, remaining colonic length, gestational age, absence of IFALD and underlying diagnosis have consistently emerged as important determinants, with NEC generally associated with more favorable outcomes and gastroschisis with poorer prognosis [108,109,110,111,112]. Additional factors such as higher serum citrulline levels, better enteral tolerance at six months post-resection, and consistent multidisciplinary follow-up have been linked to PN discontinuation [112]. Importantly, these associations derive predominantly from retrospective studies, and their predictive value at the individual patient level remains limited. Psychosocial factors, including strong caregiver–provider relationships and adherence to nutritional recommendations, appear to play a meaningful role in PN weaning success [36]; however, these clinically relevant factors are difficult to quantify and are therefore underrepresented in existing prognostic models, despite their importance in everyday clinical decision-making.

Several tools have been proposed to better quantify intestinal sufficiency and predict PN weaning. The PN Dependence Index (PNDI), defined as the ratio of non-protein energy delivered by PN to resting energy expenditure using Schofield equations, has been suggested to provide a more informative measure than enteral intake alone [113]. Although thresholds have been proposed to categorize PN dependence—where a PNDI > 120% indicates very high PN dependence, 80–120% high dependence, and <80% mild dependence—these cutoffs have not been prospectively validated [114]. Combining PNDI with serial citrulline measurements may improve predictive accuracy [115], although this approach has not been systematically tested across diverse SBS populations. Similarly, the SBS severity score developed by Belza et al., which integrates small-bowel length, ICV status, conjugated bilirubin concentration, and enteral intake at six months postoperatively, has shown associations with EA achievement within 24 months [116,117]. While promising, these tools are derived from limited cohorts and should be interpreted as risk-stratification aids rather than definitive predictors.

#### 4.3.1. Stepwise Weaning and Feeding Advancement

Approaches to PN weaning and enteral advancement also vary substantially among centers. EN is typically advanced gradually, with careful monitoring of tolerance, growth trajectories, and biochemical stability. Tolerance is generally defined by the ability to increase enteral or oral intake without clinically significant vomiting, diarrhea, or other signs of malabsorption [118]. Laboratory monitoring focuses on electrolytes, renal function, glucose homeostasis, and micronutrient status [111]. Throughout the transition, close, individualized follow-up by a multidisciplinary intestinal rehabilitation team is essential to balance nutritional advancement with metabolic stability and to respond promptly to emerging complications [27,111,119]. Despite the central importance of this phase, no pediatric studies have directly compared different PN weaning strategies, such as reducing the number of PN days per week [109,120], versus shortening daily infusion duration. Adult data suggest that reducing PN days may improve quality of life and lower long-term complication rates, but may also increase the risk of dehydration, electrolyte disturbances, and nutritional deficits if enteral intake is insufficient [121,122]. Extrapolation of these findings to children should therefore be approached with caution.

#### 4.3.2. Pharmacologic Support for PN Weaning

Pharmacologic adjuncts are frequently used to support nutritional management and facilitate PN weaning, including antisecretory agents (e.g., clonidine), antimotility medications (e.g., loperamide), prokinetics for delayed gastric emptying or motility disorders (e.g., in gastroschisis), and antibiotics for small-bowel bacterial overgrowth. Teduglutide, a GLP-2 analogue, has demonstrated the ability to enhance intestinal adaptation, reduce fluid losses, and decrease PN requirements [36,123]. Nevertheless, limitations related to access, cost, and long-term safety considerations limit its widespread use, and its role should be viewed as adjunctive within a multidisciplinary intestinal rehabilitation framework, rather than as a substitute for individualized nutritional optimization.

#### 4.3.3. Oral Aversion

Feeding-related challenges extend beyond nutrient delivery. Oral aversion is common in toddlers with SBS, particularly among those dependent on enteral tube feeding (ETF). Caregivers may be reluctant to introduce new foods—particularly fruits—because of concerns about feeding intolerance, while prolonged hospitalizations and limited shared mealtime experiences can further restrict typical social and developmental feeding opportunities [124]. Together, these factors may contribute to reduced dietary variety and hinder exposure to age-appropriate textures. Observational studies have reported associations between early reliance on ETF or early prolonged periods of nil per os (NPO) and delayed acquisition of feeding skills in children with SBS-IF. In addition, delayed introduction of solid foods has been associated with an increased risk of oral aversion [111,125]. In contrast, oral feeding supports development of feeding skills, hunger-satiety regulation and positive feeding experiences, underscoring the importance of timely and developmentally appropriate oral exposure when feasible [111].

Successful transition to enteral autonomy, therefore, requires not only nutritional adequacy but also structured support for feeding behavior. Family involvement, early safe oral experiences, oral-motor and sensorimotor interventions, developmentally appropriate food exposure, shared mealtimes, and flexible PN/EN scheduling to allow participation in daily activities are all important components [117]. Implementation depends on coordinated multidisciplinary care [124].

#### 4.3.4. Nutritional Status Monitoring

Even after PN discontinuation, children with SBS remain highly vulnerable to micronutrient deficiencies. Deficits in magnesium, calcium, fat-soluble vitamins (A, D, E, K), vitamin B_12_, iron, zinc, selenium and copper are frequently reported following PN weaning, with iron deficiency affecting as high as 84% during transition and persisting in up to 61% of children on full enteral nutrition [83,126,127,128]. In a cohort of 30 children with IF (90% with SBS), 70% had at least one vitamin deficiency and nearly 80% had at least one mineral deficiency after achieving enteral autonomy, with vitamin D, zinc, and iron most frequently affected (68%, 67% and 37%, respectively). Multivitamin supplementation and preservation of the ICV were identified as protective factors [129]. Reported rates of fat-soluble vitamin deficiencies remain high after weaning, with vitamin A deficiency up to 94%, vitamin E up to 61%, and vitamin D up to 59%. Trace element deficiencies—particularly zinc, copper, and selenium—are also prevalent [84,130]. Medication exposure, especially long-term proton pump inhibitors, has been associated with increased risk of copper, iron, and vitamin B12 deficiencies due to impaired gastric acid-mediated absorption, and should be critically reassessed in the long-term management of children with SBS.

Given this persistent burden of deficiencies, micronutrient monitoring is essential, particularly in light of the high risk of metabolic bone disease (MBD) in SBS, which often persists beyond PN weaning [39]. MBD results from the combined effects of malabsorption, chronic micronutrient deficiencies, inflammation, metabolic acidosis, and prolonged PN exposure, leading to impaired absorption of calcium, magnesium, phosphate, and vitamins D and K. Small intestinal bacterial overgrowth may further exacerbate bone loss through inflammatory mechanisms [131]. Accordingly, regular monitoring of bone-related markers—including vitamin D, calcium, phosphorus, alkaline phosphatase, parathyroid hormone, and urinary calcium—together with annual bone density assessment, is warranted.

Essential fatty acid deficiency may also occur during PN weaning [132], and warrants monitoring of triene/tetraene ratio. Consequently, close clinical, biochemical, and nutritional surveillance remains essential throughout PN weaning and long after PN cessation. Current ASPEN guidelines emphasize individualized monitoring in the absence of standardized evidence-based protocols [1], And the ESPGHAN position paper suggests vitamin and micronutrient levels every 6–12 months; however, in our clinical practice vitamins and micronutrients are monitored more frequently (approximately every 3 months) during PN dependence, with monitoring intervals extended only after enteral autonomy is achieved and nutritional status has stabilized.

#### 4.3.5. Hydration Monitoring

Caregivers should be taught how to track stool patterns and recognize early signs of dehydration—such as increased thirst, seeking extra fluids or salt, dark urine, fewer wet diapers, irritability, confusion, weakness, dizziness, fatigue, or excessive sleepiness [36].

Despite substantial advances in multidisciplinary care, evidence guiding optimal PN weaning strategies remains limited. Although mortality and intestinal transplantation rates have declined, the proportion of children achieving full EN has not increased substantially. A recent multicenter retrospective study of 443 pediatric patients demonstrated an improved survival and reduced transplantation rates without a corresponding increase in EA attainment, indicating that a growing number of children remain PN dependent [9]. These findings highlight persistent gaps in current management strategies and underscore the need for innovative, evidence-driven approaches to promote enteral autonomy and improve long-term outcomes in pediatric SBS.

## 5. Conclusions

The nutritional management of SBS in children requires an individualized, dynamic, and multidisciplinary approach that considers residual intestinal anatomy, the stage of adaptation, and associated comorbidities. Although advances in PN, early enteral nutrition, and dietary strategies have significantly improved clinical outcomes and survival, most current recommendations are based on observational evidence and expert consensus. Thus, clinical decisions must balance available evidence, specialized experience, and continuous monitoring of growth, nutritional status, and metabolic complications.

Enteral nutrition remains a cornerstone of management in children with SBS. Multicenter, longitudinal studies are needed to strengthen the evidence base, refine therapeutic strategies, support centralized management, improve patient outcomes, and address existing knowledge gaps.

## 6. Limitations and Future Perspectives

### 6.1. Limitations

This review has several limitations. First, most of the available evidence informing nutritional management in pediatric SBS is derived from retrospective studies, small prospective cohorts, case series, and expert consensus, with a clear scarcity of randomized controlled trials in pediatric populations. As a result, many conclusions are based on observational data and clinical experience rather than high-level evidence.

Second, the included studies are heterogeneous with respect to patient age, underlying diagnosis, extent and anatomy of bowel resection, and clinical outcomes, which limits direct comparison across studies and precludes formal quantitative synthesis. In many reports, outcomes are not consistently stratified according to key anatomical factors, such as residual bowel length, presence of the ileocecal valve, or colonic continuity.

Third, nutritional interventions varied widely in terms of feeding routes, modalities, diet composition, and advancement protocols, reflecting differences in institutional practice and limiting generalizability. In addition, although multiple major bibliographic databases were searched, EMBASE could not be included because access was not available at the time of the review; therefore, it is possible that some relevant studies indexed exclusively in EMBASE were not captured. Finally, although a broad time frame was necessary to capture the limited pediatric literature, changes in surgical techniques, nutritional formulations, and intestinal rehabilitation practices over time may influence the applicability of older studies to current clinical care.

### 6.2. Future Perspectives

Future investigations should focus on prospective, multicenter studies that assess targeted nutritional interventions across the various anatomical subtypes of pediatric SBS. Controlled trials are required to determine the optimal enteral diet composition and to better define its contribution to intestinal adaptation. The identification of reliable biomarkers predictive of enteral autonomy could support more personalized nutritional management. Moreover, long-term follow-up studies are needed to evaluate the effects of nutritional strategies on growth, neurodevelopment, and quality of life. The complex interplay between dietary interventions and gut microbiota represents an additional important area for future research. Approaches based on whole foods and blended diets warrant further validation, and the use of standardized clinical assessment tools should be encouraged. Together, these efforts will enhance the evidence base and inform best practices in pediatric intestinal rehabilitation.

## Figures and Tables

**Table 1:** Inclusion and Exclusion Criteria According to the PICOS Model.

PICOS Category	Inclusion Criteria	Exclusion Criteria
**P (Population)**	Neonates, infants, and older children with SBS	Children with intestinal failure due to motility disorders; children with SBS > 5 years of follow-up
**I (Intervention)**	Full-text papers, including RCTs, prospective cohort, analytical cross-sectional, case–control, longitudinal, case series, and retrospective cross-sectional studies	Studies using incompatible virtual methods; studies primarily focused on medical or surgical management of SBS
**C (Comparators)**	Studies comparing outcomes of nutritional management during different SBS phases or between anatomical SBS types	Studies comparing nutritional management in children vs. adults with SBS
**O (Outcomes)**	Survival, achievement of EA (weaning off PN), and complication rates	Incomplete results
**S (Study Design)**	Studies published in English between January 1974 and December 2024, indexed in PubMed, Scopus, Web of Science, CENTRAL, SciELO, and Google Scholar	Duplicates, conference papers, abstracts, and non-English case reports

Abbreviations: SBS, short bowel syndrome; RCT, randomized controlled study; EA, enteral autonomy; PN, parenteral nutrition; CENTRAL, Cochrane Central Register of Controlled Trials; SciELO, Scientific Electronic Library Online.

## Data Availability

The datasets used and/or analyzed during the current study are available from the corresponding author upon reasonable request.
